# Evaluating air leakage from staple line reinforcements in anatomical pulmonary resection (AIRSTOP): a prospective randomized controlled trial protocol

**DOI:** 10.1007/s11748-024-02111-0

**Published:** 2024-12-26

**Authors:** Jotaro Yusa, Kazuhisa Tanaka, Kohei Takahashi, Yuki Shiko, Takeshi Sugawara, Ichiro Yoshino, Hidemi Suzuki

**Affiliations:** 1 Department of General Thoracic Surgery, Chiba University Graduate School of Medicine, 1-8-1 Inohana, Chiba, 260-8670 Japan; 2 Biostatistics Section, Clinical Research Center, Chiba University Hospital, Chiba, Japan; 3 Department of Biostatistics, Graduate School of Medicine, Saitama Medical University, Saitama, Japan; 4 Clinical Research Center, Chiba University Graduate School of Medicine, Chiba, Japan; 5 Department of Thoracic Surgery, International University Health and Welfare School of Medicine, Narita, Japan

**Keywords:** Air leakage, Anatomical pulmonary resection, Staple line reinforcement

## Abstract

**Background:**

Air leakage during pulmonary resection is a major complication in thoracic surgery. It frequently occurs at sites of adhesion dissection, due to lung manipulation, and along the staple lines of automatic suturing devices, particularly in cases of pulmonary fragility such as those of emphysema and interstitial pneumonia. Persistent postoperative air leakage prolongs chest tube indwelling and extends hospitalization time. Staplers with absorbable tissue reinforcements have been introduced for pulmonary resection to prevent intraoperative stapler-related air leakage. This phase II prospective, open-label, randomized, parallel-group trial aims to validate the efficacy of staplers with or without absorbable tissue reinforcements in controlling stapler-related air leakage during anatomical pulmonary resections.

**Methods:**

Overall, 120 patients will be randomized into two groups: one that will undergo conventional anatomical pulmonary resection and the other in which staplers with absorbable tissue reinforcements will be used. The primary endpoint will be intraoperative stapler-related air leakage. Data will be analyzed between 2024 and 2025.

**Discussion:**

This trial will validate the effectiveness and safety of staple line reinforcements in controlling intraoperative air leakage during anatomical pulmonary resections, potentially leading to optimized strategies for patients with conditions such as emphysema and interstitial pneumonia.

**Trial registration:**

This trial has been registered with the Japan Registry of Clinical Trials 1032220620 (https://jrct.niph.go.jp/latest-detail/jRCTs031230224).

## Introduction

### Background

Air leakage is a common postoperative complication of anatomical pulmonary resection, often resulting from lung injury during intraoperative manipulation [1, 2]. It is particularly prevalent at sites of adhesion dissection and along the staple lines of automatic suturing devices [3].

### Rationale and knowledge gap

Staplers with absorbable tissue reinforcements, such as those made from polyglycolic acid or glycolic acid–lactic acid polyester, have been introduced to reduce air leakage along staple lines [4–7]. This innovation is anticipated to reduce intraoperative air leakage, thereby shortening the time required for repair, chest tube replacement duration, and overall hospital stay. However, the superiority of staplers with absorbable tissue reinforcement over conventional staplers has not been systematically evaluated.

### Objective

This trial aims to assess the efficacy and safety of staplers with absorbable tissue reinforcement for controlling intraoperative and postoperative air leakage during anatomical pulmonary resection.

## Methods

### Study design and objectives

#### Preoperative findings

**Fig. 1:**
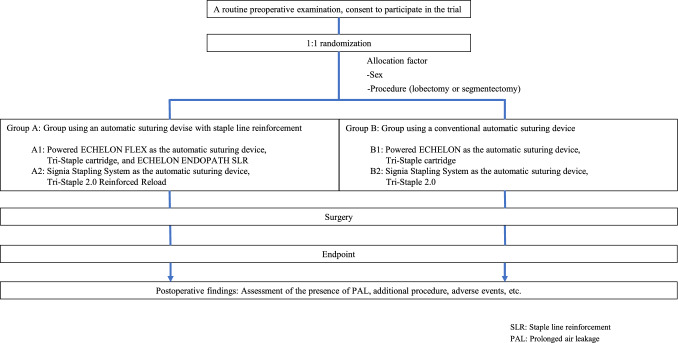
Design of the clinical trial

This prospective, randomized controlled trial will include patients with pulmonary diseases who require anatomical pulmonary resection. Routine preoperative examinations will be performed to confirm patient eligibility, and eligible patients will be required to provide informed consent. The study schema is presented in Fig. 1, and the measurement schedule is summarized in Table 1.

**Table 1:** Detailed measurement schedule

	Enrollment	Observation period		
		Operative day	After operation	Follow-up (until POD 42)
Informed consent	●			
Clinical findings	●	●	●	●
Background information	●			
Initial vital signs	●			
Spirometry	●			
Complete blood count, Biochemical testing	●		●	●
Chest X-ray	●	●	●	●
Chest CT	●			
Presence of intraoperative stapler-related air leakage (Primary endpoint)		●		
Time to repair intraoperative air leakage (Secondary endpoint)		●		
Number of automated suturing devices used in pulmonary resection (Secondary endpoint)		●		
Air leakage, due to surgical procedures (Secondary endpoint)		●	●	●
Duration of postoperative chest drain tube placement (Secondary endpoint)			●	
Presence of postoperative prolonged air leakage (Secondary endpoint)			●	
Presence of additional procedures (Secondary endpoint)			●	●
Presence of delayed air leakage (Secondary endpoint)			●	●
Postoperative adverse event (Secondary endpoint)		●	●	●

A black dot indicates a mandatory survey

*POD* Postoperative day, *Biochemical testing* measurement of glycosylated hemoglobin, albumin, and Krebs von den Lungen 6, *CT* Computed tomography

##### Inclusion criteria

1. Patients aged 18 years or older who were scheduled to undergo anatomical pulmonary resection (lobectomy or segmentectomy) using an automatic suturing device;
2. Patients with incomplete interlobar fissures on preoperative chest computed tomography (CT) who were considered to require pulmonary resection involving the use of an automatic suturing device;
3. Patients with preserved main organ function and an Eastern Cooperative Oncology Group (ECOG) performance status of 0–1; and
4. Patients fully informed of the study and who provide informed and voluntary written consent for participation.

##### Exclusion criteria

1. Patients undergoing pulmonary partial resection or requiring multiple lobectomy or segmentectomy;
2. Patients who were considered to have complete interlobar fissures on preoperative chest CT and not to require pulmonary resection involving the use of an automatic suturing device;
3. Patients with dementia or other conditions that require consent from their legally authorized representatives; and
4. Patients with known allergies to the study materials or deemed by the investigator to be unsuitable for safe participation in this study.

Incomplete interlobar fissures refer to a situation where the fissures (natural separations between lobes) in the lungs are not fully formed or do not completely divide the lung lobes.

#### Sample size

A total of 120 patients will be recruited. The recruitment period extends from January 2023 to December 2025. The presence or absence of intraoperative air leakage in the staple line reinforcement and non-use groups will be investigated. Based on the results of a retrospective study conducted at our institution, the expected intraoperative air leakage rates in the staple line reinforcement group and group without staple line reinforcement are 27% and 50%, respectively. With a significance level of 5%, power of 70%, and a 1:1 allocation between the groups, 55 patients have been estimated to be required in each group. Considering ineligible cases and dropouts, the enrollment of 60 patients in each group has been planned.

#### Participants and randomization

The patients will be enrolled before surgery and randomly allocated to four groups. The allocation process is based on the use of an automatic suturing device equipped with staple line reinforcements (absorbable tissue reinforcement composed of polyglycolic acid or glycolic acid–lactic acid polyester) versus a conventional automatic suturing device. Sex and procedure (lobectomy or segmentectomy) will be used for allocation to the following four groups: These factors have been selected because factors confounding sex such as respiratory function, smoking history, and general frailty, may influence lung fragility, and the number of automatic suturing devices planned for use may differ according to the procedure. Random allocation will be performed at a 1:1 ratio, with central registration at an independent data center, to minimize selection bias. The schematic of the study is shown in Fig. 1. Allocation for groups A and B will be achieved based on the aforementioned factors, whereas that for subgroups A1, A2, B1, and B2 was random and equal. The following devices were used in the groups:

Group A: Automatic suturing device with staple line reinforcement;

Subgroup A1: Powered ECHELON FLEX as the automatic suturing device, Tri-Staple cartridge, and ECHELON ENDOPATH^®^ Staple Line Reinforcement (ECHICON Johnson & Johnson, NY, U.S.A.) used for absorbable tissue reinforcement;

Subgroup A2: Signia Stapling System as the automatic suturing device and Tri-Staple 2.0 Reinforced Reload^®^ (Medtronic, Dublin, Ireland) used for absorbable tissue reinforcement;

Group B: Conventional automatic suturing device;

Subgroup B1: Powered ECHELON FLEX as automatic suturing device and Tri-Staple cartridge; and

Subgroup B2: Signia Stapling System as the automatic suturing device and Tri-Staple2.0.

The appropriate stapler size will be selected by the surgeon, based on the thickness and rigidity of the lung tissue to be resected.

### Procedure

#### Intraoperative findings

Factors such as background lung (presence of emphysema or interstitial pneumonia), presence of adhesions, operative time, blood loss, presence of interoperative air leakage, and time to repair pulmonary air leakage, will be assessed. Surgical cases in which the use of an automatic suturing device is judged to be not required for pulmonary resection and those considered unsuitable for en bloc resection were considered ineligible.

After pulmonary resection, a standard water-seal test will be conducted, beginning at an airway pressure of 15 cm H₂O and progressing to a maximum pressure of 20 cm H₂O. The presence, location, and extent of air leakage due to intraoperative manipulation, such as that at the staple line site, will be assessed. If air leakage is observed, its location and extent will be documented and the time required for repair (in minutes) will be recorded.

#### Postoperative findings

Postoperative assessments will focus on the presence of air leakage, duration of chest tube indwelling, and incidence of prolonged air leakage. Additionally, the need for chest tube reinsertion due to delayed air leakage following tube removal, the necessity of any further interventions, and the occurrence of any adverse events will be evaluated.

### Ethical aspects

The study will be conducted in accordance with the Declaration of Helsinki (revised in 2013).

This study has received ethical approval from the Chiba University Certified Clinical Research Review Board (CRB3180015) and has been registered with the Japan Registry of Clinical Trials (jRCT1032220620). The investigators will explain the concept of the trial to the patients and obtain written informed consent from each patients, including consent for the use of their data in study publications. The initial version of the protocol was approved on January 18th, 2023, and patient recruitment began in March 2023. The latest version 1.3, was approved on March 24th, 2024.

### Endpoints

The primary endpoint is the incidence of intraoperative stapler-related air leakage. Air leakage assessed in this study will be categorized as follows: stapler-related air leakage and air leakage not caused by the stapler. Stapler-related air leakage is defined as air leakage that is considered to have been caused by invasion with an automatic suturing device during pulmonary resection (e.g., staple holes, stamps, staple line overlapping sites, and pleural defects near the staple lines). Air leakage not caused by a stapler is defined as air leakage not caused by an automatic suturing device (e.g., pleural defects due to adhesion dissection and lung injury grasping during surgery).

The secondary endpoints are as follows: (1) time required to repair the intraoperative air leakage; (2) duration of postoperative chest tube placement; (3) use of additional procedures; (4) incidence of delayed air leakage; (5) incidence of intraoperative air leakage due to automatic suturing devices; (6) incidence of intraoperative air leakage, postoperative air leakage, and delayed air leakage during surgical procedures; and (7) number of automatic suturing devices used in pulmonary resection.

Other postoperative adverse outcomes will be confirmed as the safety endpoint.

The exploratory issues are as follows: (1) preoperative clinical diagnosis; (2) pathological diagnosis; (3) comorbidity; (4) intraoperative approach (video-assisted thoracic surgery (VATS), hybrid VATS, and open thoracotomy); (5) surgical duration (min); (6) method of the procedure if the procedure was performed for a persistent air leakage; (7) duration of postoperative hospital stay (days); (8) concomitant medications; (9) respiratory function tests; and (10) evaluation of emphysema by measuring the low attenuation volume in lung field analysis using 3D-CT images.

### Statistical analysis

For the primary endpoint, proportions were calculated and comparisons between groups were performed using the chi-square test. To supplement the analysis of the primary endpoints, we also analyzed the secondary endpoints. No multiplicity adjustments were made in the analysis of secondary endpoints. For each secondary endpoint, group comparisons were conducted using a *t* test for continuous variables and the chi-square test or Fisher's exact test for categorical variables.

The safety endpoint was the frequency of adverse events; we recorded the frequency and proportion of adverse events, created a list of these, and calculated the frequency. All comparisons were planned and all *p* values were two sided. Statistical significance was set at *P* < 0.05. All statistical analyses were performed using the SAS software (version 9.4; SAS).

### Quality control

An independent data-monitoring committee will be established for this study. The monitoring manager will be responsible for ensuring that the human rights of the subjects are protected, the study is conducted in accordance with the protocol, and the data are accurately collected. An independent data monitoring committee will perform safety monitoring, including a comparison of the incidence of adverse events in the study and a detailed review of serious adverse events as necessary, with the aim of ensuring patient safety. Occasionally, based on the results, the committee may recommend changes to the study design, such as changes in the inclusion criteria, to reduce the risk of adverse events or decide whether to continue the study. The principal investigator will decide to discontinue the study as a whole if it is considered difficult to continue because of unforeseen adverse events, illness, or other factors.

## Discussion

In general, air leakage as a complication of lung resection is related to a combination of factors such as emphysema, smoking, background lung fragility, and intraoperative manipulation [2]. The principal site for intraoperative air leakage is along the staple line, and it has been posited that the use of automatic suturing devices with staple line reinforcement can mitigate stapler-related air leakage [8]. However, previous studies have not compared the effectiveness of conventional automatic suturing devices with that of staple line reinforcement in controlling air leakage during pulmonary resection. This study aims to verify the efficacy and safety of staple line reinforcement during pulmonary resection. By identifying intraoperative factors such as the surgical procedure performed, along with patient-specific factors including background lung conditions, respiratory function, and smoking history, it may be possible to predict the occurrence of intraoperative air leakage. The judicious use of automatic suturing devices with staple line reinforcement in suitable cases can significantly reduce intraoperative air leakage, minimize the operative time associated with air leak repair, and effectively control postoperative air leakage.

## Data Availability

The datasets generated and analyzed during the current study are available from the corresponding author on reasonable request.
